# An online vignette experiment on stigma and help-seeking attitudes towards five mental health problems in adolescents and emerging adults

**DOI:** 10.1038/s41598-025-11315-0

**Published:** 2025-08-15

**Authors:** Diana Lemmer, Markus Moessner, Nicolas Arnaud, Harald Baumeister, Agnes Mutter, Sarah-Lena Klemm, Elisa König, Paul Plener, Christine Rummel-Kluge, Rainer Thomasius, Michael Kaess, Stephanie Bauer

**Affiliations:** 1 Center for Psychotherapy Research, University Hospital Heidelberg, Bergheimer Str. 54, 69115 Heidelberg, Germany; 2 Ruprecht- Karls University Heidelberg, Heidelberg, Germany; 3 German Centre for Addiction Research in Childhood and Adolescence, University Medical Centre Hamburg-Eppendorf, Hamburg, Germany; 4 Department of Clinical Psychology and Psychotherapy, Ulm University, Ulm, Germany; 5 Department of Psychiatry and Psychotherapy, University of Leipzig Medical Center, Leipzig, Germany; 6 Department of Child and Adolescent Psychiatry, Psychosomatics, and Psychotherapy, University Hospital Ulm, Ulm, Germany; 7 Department of Child and Adolescent Psychiatry, Medical University of Vienna, Vienna, Austria; 8 University Hospital of Child and Adolescent Psychiatry and Psychotherapy, University of Bern, Bern, Switzerland; 9 Clinic of Child and Adolescent Psychiatry, Center for Psychosocial Medicine, University Hospital Heidelberg, Heidelberg, Germany; 10 German Center for Mental Health (DZPG), Partner site Mannheim/Heidelberg/Ulm, Ulm, Germany

**Keywords:** Mental health, Stigma, Help-seeking, Online experiment, Vignette study, Public health, Addiction, Anxiety, Depression, Psychology

## Abstract

Public attitudes vary across mental health (MH) problems. However, research on young people and certain MH conditions is limited. This online-experiment examined stigma and potential help-seeking among 554 adolescents and emerging adults aged 14–29 years towards generalized anxiety disorder, depression (DEP), bulimia nervosa (BN), non-suicidal self-injury (NSSI), and problematic alcohol use (ALC). Participants were randomized to a video vignette depicting one of the five MH problems. Attitudes were measured with the Universal Stigma Scale (subscales: “blame/ personal responsibility” and “impairment/ distrust”) and the General Help Seeking Questionnaire assessing the likelihoods of seeking professional, informal, and no help for the respective MH problem. Data were analysed using Kruskal-Wallis and Bonferroni-corrected Dunn’s tests. Compared to all of the other conditions, ALC was the most stigmatized. Furthermore, ALC was more likely to prompt any help-seeking as compared to DEP, BN, and NSSI, and professional help-seeking in comparison to DEP. BN elicited more blame than DEP, whereas the reverse pattern emerged for distrust. However, this sample generally held positive MH attitudes. The results highlight the importance of addressing disorder-specific stigma and may inform the development of targeted anti-stigma and help-seeking campaigns.

**Trial registration**: This study has been registered at the German Clinical Trials Register (www.drks.de) on September 23rd, 2020 https//drks.de/search/de/trial/DRKS00023110 #DRKS00023110.

**Supplementary Information:**

The online version contains supplementary material available at 10.1038/s41598-025-11315-0.

## Introduction

With at least 10% of children and adolescents^1,2^ and 25–39% of college students^3,4^ affected by common mental health (MH) problems such as anxiety and depression, MH problems in youth are increasingly prevalent and pose high burdens on both individuals and societies^5,6^. Approximately half of all mental disorders first develop by the age of 18 years^7^ and they frequently persist into adulthood^8^. While effective treatment exists, large proportions of young people either do not seek any support or receive treatment after substantial delays^9,10^, resulting in an alarming rate of unmet MH care needs of almost 60%^11^. Attitudinal barriers such as stigmatizing beliefs and a low MH literacy^12–14^ largely contribute to delayed help-seeking and treatment uptake. Previous literature has identified various aspects of stigma, manifesting on systemic, behavioural, and attitudinal levels as stereotypes and prejudice (i.e. beliefs or cognitive responses that associate undesirable characteristics with MH problems) as well as discrimination (i.e. social avoidance, distancing, and exclusion on interpersonal and structural levels)^15^. While public stigma in particular encompasses this variety of behavioural, systemic, affective, and cognitive aspects as well, on an attitudinal level, it refers to the agreement with negative stereotypes of the general population towards individuals with MH conditions^16^. While personal attitudes towards MH problems have previously also been termed “personal stigma” as opposed to “perceived (public) stigma”, i.e. the awareness of public stigma in the general population^17^, the term “public stigma” is used in the following to maintain consistency. A deeper understanding of these attitudinal barriers and the development of effective strategies to overcome them constitute major public health objectives^15^.

While stigmatizing beliefs affect MH problems in general, previous studies have identified differences in stigma across specific MH problems. These studies frequently utilized case vignettes, i.e. diagnostic descriptions of individuals affected by selected sets of symptoms, which provide more nuanced stimuli as compared to simple labels and can be used in experimental contexts via random assignment^18^. For instance, a representative Swiss vignette survey with an adult sample demonstrated that alcohol dependency symptoms and other-endangering behaviours increased the perceived dangerousness (i.e. perceptions of unpredictability and violence) of vignette characters^19^. A similar pattern was found in a study with Australian adolescents, wherein a vignette peer who displayed alcohol misuse was perceived as both more dangerous and as “weak” rather than sick in comparison to a depressed vignette character^20^. Both stigma dimensions, dangerousness and the “weak-not-sick” facet, were associated with weaker intentions to encourage informal help-seeking (e.g. consulting friends or family members), while dangerousness was associated with stronger intentions to recommend formal help-seeking (e.g. consulting MH professionals). Other studies compared stigmatizing attitudes among college students towards vignette characters depicting either obesity, eating disorders, or depression. While characters with obesity were blamed (i.e. held responsible) more strongly for their condition than characters with eating disorders, which in turn were blamed more than characters with depression, depression was associated with higher perceived impairment and distrust^21,22^. However, most studies addressed adult samples and a limited amount of MH problems, such as general MH, depression, schizophrenia^18,23,24^ or attention deficit disorder in the field of children and adolescents^25^. Youth samples and other MH problems such as eating disorders are less represented. As previous studies suggest differences in stigmatizing attitudes in relation to age^26,27, ^gaining a deeper understanding of stigma in different phases of life is crucial for the development of targeted anti-stigma interventions.

### Current study

This secondary analysis of an online-experiment examined and compared public stigma (other-oriented) and potential help-seeking (self-oriented) among adolescents and emerging adults aged 14 to 29 years between five MH problems, namely generalized anxiety disorder (GAD), depression (DEP), bulimia nervosa (BN), non-suicidal self-injury (NSSI), and problematic alcohol use (ALC), expanding previous research by including MH conditions that have been less studied in young people. This study followed an exploratory approach and did not test specific hypotheses.

## Methods

### Study design

This study is based on a secondary analysis of data from a larger online-experiment with 15 conditions in total (5 MH problems × 3 interventional conditions). The primary study compared the effects of two video-based micro-interventions against a vignette-only control group (see Lemmer et al. (2024)^28^ for detailed information). While the two intervention groups were presented with an additional video aiming to either induce positive outcome-expectancies of help-seeking or to provide psychoeducational and destigmatizing information, only the vignette video which is described in more detail below was shown to the control group. Group allocation was implemented through an automated permuted block randomization (block sizes: 15, 30), stratified by gender. Participants who identified as men/ boys were presented with a male protagonist (“Paul”), while participants who identified as women/ girls and participants with another gender identity viewed a female protagonist (“Paula”). The current study uses only data from the five control conditions to ensure comparability, as differential intervention effects were found across MH conditions in the primary study. Allocation to the five groups was randomized.

### Recruitment and sample

Ethical approval was obtained from the Ethics Committee I of the Heidelberg Medical Faculty on 27th of July, 2020, protocol number S-378/2020. Adolescents and emerging adults aged 14 to 29 years with sufficient German language skills were recruited between October 2020 and May 2022. The age limit of 14 years was selected due to its widely accepted adequacy for the provision of informed consent in health-related decisions and study participation^29,30 ^while the upper age limit of 29 years conforms with the age span of emerging adulthood, a distinct life stage between adolescence and established adulthood^31,32^. A convenience sample was primarily recruited online via social media, mailing lists, online forums, and online market places targeted at youths (e.g. online groups, accounts, and mailing lists of youth clubs or student associations). In an optional gift card lottery at the end of the study, participants had the chance to win one of 100 gift cards à 20 €. Participants who voluntarily provided their e-mail addresses at the end of the survey were eligible for the lottery. E-mail addresses were stored separately from other participant data to ensure anonymity.

**Fig. 1:**
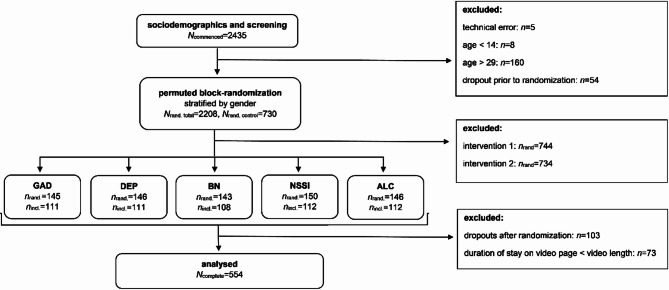
Study design and sample sizes.

Figure 1 shows the flow of participants. In total, 730 participants who fulfilled the inclusion criteria were randomized to one of the five control conditions. Participants who did not complete data assessment (*n* = 103) and participants whose duration of stay on the video page fell below the length of the respective video they were assigned to watch, as indicated by page change timestamps, were excluded (*n* = 73). The final sample included *N* = 554 young persons (75.89%).

*n*_rand_. = randomized cases: number of randomized control group cases, irrespective of data completeness and video page duration of stay; *n*_incl_. = included cases: number of control group cases with complete data and sufficient durations of stay on the video vignette page which were included in the data analysis.

### Procedure

This web-based experiment was implemented in the software ASMO^33^. It was openly accessible via a website containing the study information. Participants received extensive information about the study aims, procedures, data processing, and data storage prior to their participation. However, the information did not specify the five MH problems of the study. Instead, the general term “health problems” was used to avoid self-selection biases. After providing informed consent through an online checkbox, participants were presented with sociodemographic and screening questionnaires, watched the randomly assigned video, completed outcome questionnaires, and received MH emergency contact information on a debriefing page. Once the participants had confirmed their responses through a click on the “next”-button, they were not able to change the responses. There was no “back”-button. All items had to be completed in order to proceed with the study. In total, this study took participants approximately 30 min to complete. The study was conducted in German.

### Measures

#### Sociodemographics and screening

All measures were self-reported assessments used to describe the characteristics of the sample. The sociodemographic form included questions about the participants’ age, gender, migration background, education, exposure to someone affected by MH problems (one item: “Do you know someone who has a mental illness? Similar terms are mental health problems, emotional problems, psychological issues, mental disorders, and personal difficulties.”), and their personal MH service use (past or current actual help-seeking).

The Generalized Anxiety Disorder Scale (GAD-7)^34^ was used to assess anxiety symptoms (current sample Cronbach’s α = 0.88; McDonald’s ω = 0.88). Across its seven items, symptom frequencies were measured on a 4-point response scale. Sum scores ≥ 5 indicate mild, ≥ 10 moderate, and ≥ 15 severe anxiety symptoms (potential range: 0–21)^31^.

The 9-item version of the Patient Health Questionnaire (PHQ-9)^35^ was used to measure depression symptoms (current sample Cronbach’s α = 0.88; McDonald’s ω = 0.88). Symptom frequencies within the past two weeks were indicated on a 4-point scale. Sum scores ≥ 5 are interpreted as mild, ≥ 10 as moderate, ≥ 15 as moderately severe, and ≥ 20 as severe depressive symptomatology (potential range: 0–27)^35^.

The Weight Concerns Scale (WCS)^36,37^ is a five item questionnaire measuring weight and body shape concerns (current sample Cronbach’s α = 0.86; McDonald’s ω = 0.87). Scores ≥ 57 indicate an elevated risk for eating disorders (potential range: 0–100)^36^.

To assess NSSI, four items of the Self-Injurious Thoughts and Behaviors Interview (SITBI-G)^38^ were used (lifetime NSSI, frequency of NSSI within the last year, ages of first and last NSSI).

The Alcohol Use Disorder Identification Test-Consumption (AUDIT-C)^39,40^ screened for problematic alcohol use during the past 12 months. It consists of three items with 5-point scales (current sample Cronbach’s α = 0.72; McDonald’s ω = 0.77). A sum score of 0 indicates abstinence, scores between 1 and 3 are indicative of moderate alcohol consumption, and scores ≥ 4 are interpreted as hazardous alcohol consumption (potential range: 0–12)^39,41^.

#### Outcomes

The Universal Stigma Scale (USS)^21^ with its subscales blame/ personal responsibility (5 items; current sample Cronbach’s α = 0.75; McDonald’s ω = 0.76; example item “Paula could pull herself together if she wanted to”) and impairment/ distrust (6 items; current sample Cronbach’s α = 0.77; McDonald’s ω = 0.77; example item: “People with a problem like Paul’s are dangerous”) was applied to assess stigmatizing attitudes towards the five MH problems on a 5-point Likert scale. For each of the two subscales, means were calculated. Lower scores indicate higher stigmatization.

A 12-item version of the General Help Seeking Questionnaire (GHSQ) was used to assess the potential use of formal, informal, and no support (i.e., the hypothetical willingness to seek help if participants themselves were affected by the MH problem assigned to them) within the next four weeks^42^. The GHSQ assesses the likelihood of seeking different sources of help for a previously defined problem on a 7-point rating scale (1 = “extremely unlikely”, 7 = “extremely likely”). The participants were thus asked about their likelihoods of approaching different sources of help if they themselves were experiencing the vignette character’s problem. Help-seeking was operationalized by the maximum scores of the items representing professional (psychotherapist, psychiatrist, counselling service) and informal (friend, parent, other family member, romantic partner) sources of MH support. No intention of seeking any help was measured with one GHSQ-item (“I would not seek any help”).

Video acceptability was measured with three translated items from the acceptability/ likability scale used by Gaudiano, Davis, Miller, and Uebelacker (2020)^43^ to assess overall likability, comprehensibility, and interestingness on a 5-point rating scale (1 = “not at all”, 5 = “very”).

### Experimental conditions and materials

The experimental conditions were implemented with brief animated videos depicting case vignettes for each of the five MH problems. The vignettes were similarly structured across MH problems. First, the protagonists were introduced as 16-year old high school students in a third-person perspective. The videos then described challenges in their everyday lives due to their respective MH condition (e.g. difficult emotions and cognitions, social and school-related issues, physical symptoms). The vignettes were fully descriptive and did not include any diagnostic labels^44^. The BN vignettes were developed first and served as a template for the other four MH problems. The vignettes were inspired by Mond et al. (2010)^45^ and adapted in accordance with ICD-10 and DSM-5 diagnostic criteria as well as further literature on the respective symptomatology and psychological distress (e.g. DeJong et al., 2012^46^). Each study site developed a vignette for a MH problem that was aligned with their respective area of expertise.

Vignette durations ranged from 02:19 to 02:47 min (*M* = 02:29, *SD* = 0:11). We used the online animation tool Powtoon Pro + to create the videos. A convenience sample of *N* = 9 youths (*M*_age_ = 18.56, *SD* = 3.74, range: 14–24 years, 1/3 male) pre-tested a subset of the videos between July and September 2020 and confirmed their overall acceptability and comprehensibility.

### Statistical analysis

Sociodemographic, screening, and outcome data were first analysed descriptively. Kruskal-Wallis (KW) and chi squared tests were performed to check for group differences in the screening and video acceptability measures. Due to non-normal distributions, KW tests were conducted to test group differences between the experimental conditions (MH problems) on the primary (stigma) and secondary outcomes (potential help-seeking). Analyses of covariance (ANCOVAs) were further conducted to control for actual help-seeking, the gender of the vignette character (fixed factors), and age (covariate). However, as the ANCOVAs yielded similar results to the KW tests and the main outcome data distributions were strongly deviating from normality, indicating a better suitability of nonparametric approaches^47^only the KW test results are reported. Bonferroni-corrected Dunn’s post-hoc tests between experimental conditions were conducted whenever significant (*p* < 0.05) group main effects in the KW tests were found. η²_H_ Effect sizes are reported for the KW tests^48^. For the post-hoc comparisons, *r* coefficients were calculated as effect sizes (common cutoff-points: *r* = 0.10 for small, *r* = 0.30 for medium, *r* = 0.50 for large effects)^49,50^. Statistical analyses were conducted with SPSS 28 and R 4.3.

## Results

### Descriptive statistics

#### Sample characteristics

The participants’ age ranged between 14 and 29 years (*M* = 20.86, *SD* = 3.60). In total, 79.06% of the sample identified as women/ girls and 44.40% reported that they had sought professional help for a MH problem. Sociodemographic and screening results are displayed in Tables 1 and 2. Separate sample characteristics for each of the five vignette groups are shown in Supplementary Tables S1 to S5. On average, participants reported mild to moderate depression symptoms (PHQ-9: *M* = 9.76, *SD* = 6.16) and mild anxiety symptoms (GAD-7: *M* = 8.60, *SD* = 5.06). An elevated eating disorder risk was found in 19% of the sample, while hazardous alcohol consumption affected 31%. Lifetime and 12-month prevalences of NSSI amounted to 37% and 20%, respectively.

**Table 1:** Sample characteristics (*N* = 554).

Characteristic	Category	M (SD) or *n* (%)
Age	*M* (*SD*)	20.86 (3.60)
Gender*	Woman/ girl	438 (79.06)
	Man/ boy	102 (18.41)
	Other	14 (2.53)
Actual help-seeking	None/ never	308 (55.60)
	Current	101 (18.23)
	Past	145 (26.17)
Knowing someone with MH problems	Yes	518 (93.50)
	No	36 (6.50)
Education (highest secondary school diploma)	Still in school	111 (20.04)
	No diploma	0
	Lower secondary school diploma (Hauptschulabschluss)	3 (0.54)
	Intermediate secondary school diploma (Realschulabschluss)	36 (6.50)
	Higher/ academic secondary school diploma (Abitur)	398 (71.84)
	Other	6 (1.08)
Migration background	None	434 (78.34)
	First-generation or not from Germany	24 (4.33)
	Second-generation	96 (17.33)

*Participants were originally presented with the question “What is your gender?” and the categories “female”, “male”, and “other” in this study. To align with accurate terminology referring to gender instead of biological sex, the categories were adapted in this manuscript.

**Table 2:** Screening results and video acceptability (*N* = 554).

Measure	Category	M (SD) or *n* (%)
GAD-7	*M* (*SD*)	8.60 (5.06)
	Minimal/ no anxiety (0–4)	147 (26.53)
	Mild (5–9)	191 (34.48)
	Moderate (10–14)	133 (24.01)
	Severe (≥ 15)	83 (14.98)
PHQ-9	*M* (*SD*)	9.76 (6.16)
	Minimal/ no depression (0–4)	135 (24.37)
	Mild (5–9)	163 (29.42)
	Moderate (10–14)	130 (23.47)
	Moderately severe (15–19)	81 (14.62)
	Severe (≥ 20)	45 (8.12)
WCS	*M* (*SD*)	34.45 (24.25)
	High risk (≥ 57)	106 (19.13)
	Low risk (< 57)	448 (80.87)
SITBI-G	Lifetime NSSI	203 (36.64)
	12-month NSSI	113 (20.40)
	# of NSSI events (past 12 months) *M* (*SD*) (lifetime NSSI sample, *n* = 203)	14.46 (51.18)
	# of NSSI events (past 12 months) *M* (*SD*) (12-month NSSI sample, *n* = 113)	25.98 (66.50)
	Age first NSSI (*n* = 203)	13.83 (2.69)
	Δ age last NSSI – age first NSSI (*n* = 202)*	3.95 (3.39)
AUDIT-C	*M* (*SD*)	2.58 (2.09)
	Abstinent (0)	119 (21.48)
	Moderate (1–3)	261 (47.11)
	Hazardous (≥ 4)	174 (31.41)
Video acceptability	General likability	3.85 (0.78)
	Comprehensibility	4.79 (0.50)
	Interestingness	3.84 (0.93)

*n = 1 missing due to invalid values (age of first NSSI > age of last NSSI).

#### Video acceptability

On 5-point scales, the videos were, on average, rated as very comprehensible (*M* = 4.79, *SD* = 0.50), and moderately to highly likeable (*M* = 3.85, *SD* = 0.78) and interesting (*M* = 3.84, *SD* = 0.93). Most participants (*n* = 395, 71.30%) rated the general likability with a “4” or “5”, while only 26 individuals (4.69%) gave a rating of “1” or “2”. Almost all participants (*n* = 542, 97.83%) rated the comprehensibility with a score of “4” or “5”, with a minority of *n* = 5 (0.90%) participants assigning a score of “1” or “2”. Interestingness was rated with a “4” or “5” by *n* = 385 (69.49%) participants and with a “1” or “2” in *n* = 46 (8.30%) cases.

As indicated by KW and chi-squared tests prior to the main analysis, the groups did not differ significantly with regard to video acceptability (general likability: *H*(4) = 6.36, *p* = 0.17; comprehensiveness: *H*(4) = 8.42, *p* = 0.078; interestingness: *H*(4) = 6.37, *p* = 0.17) and all but one of the screening measures (age: *H*(4) = 2.07, *p* = 0.72; GAD-7: *H*(4) = 2.62, *p* = 0.62; PHQ-9: *H*(4) = 3.93, *p* = 0.42; WCS: *H*(4) = 2.12, *p* = 0.71; number of NSSI events during the past year: *H*(4) = 8.05, *p* = 0.090; AUDIT-C: *H*(4) = 1.48, *p* = 0.83; gender of the vignette characters: χ²(4) = 0.62, *p* = 0.96; dichotomized actual help-seeking: χ²(4) = 4.06, *p* = 0.40). The proportions of participants reporting lifetime NSSI differed significantly between the NSSI (*n* = 30, 26.79%) and the DEP groups (*n* = 52, 46.85%; χ²(4) = 10.85, *p* = 0.028; Bonferroni corrected pairwise post-hoc proportion test: χ²(4) = 9.65, *p* = 0.019). As two-factorial Puri and Sen tests (i.e. generalized KW tests^51^) which included lifetime NSSI as a second factor yielded similar results as the one-factorial KW tests, and none of the interaction effects with the MH problems were statistically significant, the one-factorial KW test results are reported. However, comparisons between the DEP and the NSSI groups should be interpreted with caution, as the significantly lower rate of individuals reporting lifetime NSSI yields contextual relevance in addition to its statistical impact.

### Differences between MH problems

Descriptive and KW outcome results are displayed in Table 3.

**Table 3:** Differences in stigma and potential help-seeking between MH problems.

	Total *N* = 554	GAD *n* = 111	DEP *n* = 111	BN *n* = 108	NSSI *n* = 112	ALC *n* = 112	H(4)	*p*	η²_H_	Pairwise comparisons
Stigma (USS)^a^										
Blame *M (SD)*	4.41 (0.68)	4.54 (0.63)	4.61 (0.63)	4.41 (0.63)	4.51 (0.59)	4.00 (0.75)	67.60	< 0.001	0.12	ALC < GAD, DEP, BN, NSSI; BN < DEP
Distrust *M (SD)*	3.84 (0.79)	4.06 (0.66)	3.86 (0.68)	4.24 (0.59)	4.00 (0.67)	3.07 (0.76)	134.81	< 0.001	0.24	ALC < GAD, DEP, BN, NSSI; BN > DEP
Potential help-seeking (GHSQ)^b^										
Professional max. *M (SD)*	4.65 (1.88)	4.49 (1.91)	4.32 (1.89)	4.62 (1.90)	4.78 (1.82)	5.05 (1.81)	11.03	0.026	0.013	ALC > DEP
Informal max. *M (SD)*	5.87 (1.37)	6.09 (1.13)	5.79 (1.45)	5.67 (1.58)	5.63 (1.50)	6.18 (1.04)	9.80	0.044	0.011	n.s.
None *M (SD)*	3.08 (2.02)	2.99 (1.93)	3.52 (2.23)	3.34 (2.11)	3.16 (1.98)	2.39 (1.68)	18.32	0.001	0.026	ALC < DEP, BN, NSSI

^a^ Higher scores represent *less* public stigma/ *more positive* attitudes towards the respective MH conditions. ^b^ Higher scores represent a higher likelihood of seeking help with the stated sources of support.

#### Stigma (USS)

In the USS-subscale blame/ personal responsibility, the KW test revealed a statistically significant group main effect (*H*(4) = 67.60, *p* < 0.001, η²_H_ = 0.12). Bonferroni-corrected post-hoc tests demonstrated a clear pattern where blame/ personal responsibility scores were significantly lower (i.e. agreement with stigmatizing statements was higher) in the ALC group as compared to any of the other conditions (GAD: *z* = 6.31, *p* < 0.001, *r* = 0.42; DEP: *z* = 7.54, *p* < 0.001, *r* = 0.50; BN: *z* = 4.28, *p* < 0.001, *r* = 0.29; NSSI: *z* = 5.64, *p* < 0.001, *r* = 0.38). Furthermore, participants in the DEP condition reported higher scores (i.e. less blame) than participants in the BN condition (*z* = 3.20, *p* = 0.014, *r* = 0.22).

With regard to the USS-subscale impairment/ distrust, a statistically significant group main effect was found (*H*(4) = 134.81, *p* < 0.001, η²_H_ = 0.24). According to the post-hoc comparisons, the mean impairment/ distrust score was significantly higher (i.e. self-reported distrust was lower) in the BN group as compared to the DEP group (*z* = 4.05, *p* = 0.001, *r* = 0.27). Moreover, impairment/ distrust scores were significantly lower (i.e. distrust was higher) in the ALC group compared to all of the other conditions (GAD: *z* = 8.73, *p* < 0.001, *r* = 0.58; DEP: *z* = 6.69, *p* < 0.001, *r* = 0.45; BN: *z* = 10.71, *p* < 0.001, *r* = 0.72; NSSI: *z* = 8.18, *p* < 0.001, *r* = 0.55).

#### Help-seeking (GHSQ)

99 participants (17.87%) indicated a low likelihood of seeking professional help (scores of “1” or “2”), whereas *n* = 216 participants (38.99%) reported a high willingness to seek professional support (scores of “6” or “7”). Potential professional help-seeking differed between the conditions (*H*(4) = 11.03, *p* = 0.026, η²_H_ = 0.013). Pairwise comparisons revealed a significant mean difference between the ALC and the DEP groups (*z* = 3.10, *p* = 0.019, *r* = 0.21).

Few participants (*n* = 20, 3.61%) reported a low likelihood of seeking informal help (scores of “1” or “2”), while the majority of the sample (*n* = 385, 69.49%) selected scores of “6” or “7”, with significant differences between the five conditions (*H*(4) = 9.80, *p* = 0.044, η²_H_ = 0.011). None of the pairwise comparisons were statistically significant. Excluding the *n* = 5 cases with a comprehensibility score of less than 3 yielded similar results. Only the group effect for informal help no longer reached statistical significance (*H*(4) = 9.04, *p* = 0.060, η²_H_ = 0.0093).

While *n* = 264 participants (47.65%) reported a low likelihood of not seeking any support (scores of “1” or “2”), *n* = 89 participants (16.06%) selected a score of “6” or “7”. Mean scores for no intention to seek any help differed significantly between the MH conditions (*H*(4) = 18.32, *p* = 0.001, η²_H_ = 0.026). Scores in the ALC group (*M* = 2.39, *SD* = 1.68) were significantly lower in comparison to the DEP (*z* = 3.89, *p* = 0.001, *r* = 0.26), BN (*z* = 3.37, *p* = 0.007, *r* = 0.23), and NSSI groups (*z* = 2.82, *p* = 0.049, *r* = 0.19).

## Discussion

### Principal findings

This study compared stigmatizing and help-seeking related attitudes towards five MH problems in a sample of *N* = 554 adolescents and emerging adults aged 14 to 29 years. In comparison to the other four MH problems, ALC clearly stands out. While ALC emerged as the most stigmatized MH problem on both public stigma dimensions (blame/ personal responsibility and impairment/ distrust), it was also associated with a higher likelihood of potentially seeking professional support as compared to DEP. Moreover, the likelihood of not seeking any support was significantly lower for ALC than for DEP, BN, and NSSI. With respect to public stigma, the results are in line with previous research demonstrating the particularly pronounced and distinct stigmatization of and discrimination against individuals with alcohol use disorder in comparison to other substance-unrelated MH problems^52,53^. However, given the well-recognized detrimental impact of stigma on help-seeking^14, ^the higher likelihoods of seeking any help and professional support for ALC in particular are surprising at first glance, even though relationships between stigma and help-seeking were not investigated in this study. On the other hand, a review by Hammarlund et al. (2018)^54^ demonstrated mixed effects of stigma on treatment-seeking decisions in alcohol- and drug-use disorders, with some studies identifying stigma as a positive predictor of help-seeking, while others found it to be a relevant barrier or observed no substantial impact. The authors argue that both the specific constructs measured, ranging from self-stigmatization to experiences of discrimination, as well as the target samples (e.g. participants with or without prior help-seeking experiences) varied considerably in this field of research, which may have contributed to heterogeneous results. The specificities of our study regarding its cross-sectional experimental design, its measures (public stigma towards others, potential help-seeking relating to oneself), and its sample composition should undoubtedly be taken into account in the interpretation of its results. Indeed, in a similar study, alcohol misuse was perceived as more dangerous than depression among a sample of high school students, with dangerousness positively predicting intentions to encourage formal help-seeking to an affected vignette peer^20^. Even though the current study measured a different facet of help-seeking (self-related instead of other-related) and investigated differences in the perception of MH problems instead of associations between stigma and help-seeking, limiting the comparability with the aforementioned study, these findings are in line with our results and might indicate a greater publicly perceived urgency of help-seeking in ALC due to the pronounced perception of dangerousness, but may not be applicable to other contexts, as we further discuss in the limitations section below.

While a significant overall group effect was found on the likelihood of seeking informal support, none of the post-hoc comparisons reached statistical significance. As the likelihood of seeking informal help was high across MH problems (all means reached a score above 5 on a 7-point scale), this result might suggest minimal overall differences which may not have been detected in the pairwise comparisons due to a low variability. Moreover, after the exclusion of *n* = 5 participants who indicated a poor comprehensibility of the vignettes, the group effect no longer reached statistical significance. While these results do not allow for specific conclusions about the MH problems included in this study, they confirm previous findings showing the importance of and preference for informal help in youths^55–57^. This emphasizes the potential of informal sources of support to either facilitate or obstruct treatment-seeking for MH problems^58^. As peer-led MH interventions have been shown effective in the promotion of adolescents’ help-seeking intentions^12^ and MH literacy was positively associated with peer-recommendations for formal help in previous research^59, ^a deeper understanding of attitudes towards informal help-seeking for specific MH problems as well as the development and dissemination of targeted psychoeducational interventions for informal supporters could be essential steps in reducing the MH treatment gap.

Additionally, more blame and personal responsibility was reported for BN in comparison to DEP, while the opposite pattern was observed in the impairment/ distrust subscale of the USS, supporting previous findings of inverse associations between these public stigma dimensions in eating disorders and DEP^21,22^. As Ebneter and Latner (2013)^21^ and Thörel et al. (2021)^22^ argue, these results may suggest an underestimated public perception of the severity of eating disorders such as BN, resulting in stronger attributions of personal responsibility. Furthermore, the behavioural aspects of eating disorders as opposed to more internalizing MH conditions such as DEP may be associated with higher perceptions of voluntariness, control, and a lack of self-discipline^21^. Conversely, an increased awareness of the severity of depression may involve stronger perceptions of distrust and, in turn, a greater inclination towards social distance. However, as our results demonstrate, this association may be limited to specific MH conditions, whereas both facets of public stigma may be similarly pronounced towards other MH problems such as ALC. This further underscores the importance of understanding and addressing MH problem specific stigmata, which this experimental study is contributing to by involving a range of different and partly understudied MH problems.

As this study aimed to investigate public stigma and potential help-seeking in a general sample of adolescents and emerging adults, it should be noted that the vignettes and measures related to them were hypothetical. They were not necessarily related to the lived experiences of the participants as we did not aim to recruit a clinical sample. As previous research has shown that adolescents are both more likely to recommend formal help to peers than seeking help for themselves^59^ and to exhibit less public stigma in comparison to self-stigma^60, ^suggesting differences in stigmatizing perceptions of oneself and others, the results of this study may not be applicable to, for instance, clinical samples experiencing stigmata related to the MH problems they are personally affected by. While the inclusion of a measure assessing potential help-seeking from the participants’ perspective served as an approximation of help-seeking intentions in a non-clinical sample, a help-recommendation measure could have been a valuable addition as it would have aligned more closely with the participants’ viewpoints as well as the public stigma measures. By acknowledging these different perspectives, future research that assesses and compares them may lead to a more comprehensive understanding of help-seeking and stigma.

Moreover, attitudes and intentions are not always accurate predictors of actual behaviour (intention-behaviour gap)^61 ^which has been observed in treatment-seeking for MH problems as well. For instance, help-seeking intentions did not significantly predict actual help-seeking at a three-year follow-up in a Swiss sample of 16- to 40-year olds with MH problems, even though a small significant correlation was found^62^. While approximately 85% of the sample initially stated an intention to seek professional help, only 20% reported actual help-seeking^62^. Furthermore, only anticipated stigma (i.e. expected embarrassment if friends were to find out about the utilization of MH services) was negatively associated with actual help-seeking, whereas no direct significant impacts were found from personal (i.e. willingness to interact with depressed or schizophrenic patients) or perceived stigma (i.e. perceptions of public opinions about MH problems), highlighting once again that close attention should be paid to specific constructs and contexts of MH stigma research. As the aim of the current study was to investigate public stigma and hypothetical help-seeking intentions, the applicability of our findings may not generalize beyond these constructs.

### Limitations

As mentioned above, the design, target sample, and actual sample composition of this study limit its applicability to other contexts. Even though we aimed to recruit a young community sample, participants generally reported low stigma and a high educational status. Furthermore, the sample included a relatively high proportion of severely distressed individuals (e.g. 8% reported severe depression symptoms as compared to approximately 2% in other studies with university students^63^), high rates of past or current help-seeking (45%), and comparably few participants identifying as men or boys (18%), which limits the generalizability of our results. As this study was openly accessible, resulting in self-selection into the study, it is plausible that young individuals with an elevated interest in MH were more inclined to participate, even though the general term “health issues” was used in the instructions instead of “MH problems” and we were careful not to specifically recruit psychology or medicine students. The sample characteristics observed in this study appear to be typical for openly accessible MH-related studies using social media as a primary recruitment channel. For instance, Lee et al. (2020)^64^ observed an overrepresentation of female, highly educated, and psychologically distressed participants who were recruited via Facebook advertisements for their suicide prevention study, which is consistent with our results. However, the use of targeted advertisements for men resulted in higher proportions of male participants, suggesting that adaptive recruitment strategies could be used in future studies to recruit more representative samples.

One further limitation of our study lies within the absence of a comprehension check to assure that the participants fully engaged with and understood the contents of the vignettes. Even though the durations of stay on the video pages were taken into account in the data analysis and the videos were rated as highly comprehensible (*M* = 4.79 on a 5-point scale), it remains unknown to what extent the participants truly attended to and comprehended the vignettes. To enhance the robustness of the findings, similar studies should include comprehension checks.

## Conclusions

The results add to previous findings on differences in public stigma across MH conditions in adolescents and emerging adults and highlight the particular stigmatization of ALC as compared to GAD, DEP, BN, and NSSI. Furthermore, BN vignettes were blamed more for their condition than DEP vignettes, while the opposite pattern was observed with respect to perceived impairment and distrust. At the same time, potential help-seeking was more likely for ALC than other MH problems, suggesting that higher public stigma may not act as a barrier to help-seeking in all contexts. The results contribute to a broader understanding of specific attitudes towards different MH conditions and can help develop and optimize targeted anti-stigma and help-seeking campaigns.

## Electronic supplementary material

Below is the link to the electronic supplementary material.

Supplementary Material 1

## Data Availability

The datasets used and analysed during the current study are available from the corresponding author on reasonable request.

## Abbreviations

ALC: Problematic alcohol use
ANCOVA: Analysis of covariance
AUDIT-C: Alcohol use disorders identification test – alcohol consumption questions
BN: Bulimia nervosa
DEP: Depression
GAD: Generalized anxiety disorder
GAD-7: Generalized anxiety disorder scale-7
GHSQ: General help-seeking questionnaire
KW: Kruskal-Wallis
MH: Mental health
NSSI: Non-suicidal self-injury
PHQ-9: Patient health questionnaire-9
SITBI-G: Self-injurious thoughts and behaviors interview – German version
USS: Universal stigma scale
WCS: Weight concerns scale
