# Supplementary material for: An online vignette experiment on stigma and help-seeking attitudes towards five mental health problems in adolescents and emerging adults
Source: Sci Rep. 2025 Aug 15;15:29956. doi: 10.1038/s41598-025-11315-0 (PMC12356931; doi:10.1038/s41598-025-11315-0)
Supplement: Supplementary file 1 — Supplementary Material 1 [file 41598_2025_11315_MOESM1_ESM.docx]

## **Supplementary Table S1. Screening results and video acceptability of participants in the Generalized Anxiety Disorder vignette group (*N* = 111)**

| Measure | Category | *M* (*SD*) or  *n* (%) |
| --- | --- | --- |
| GAD-7 |  |  |
|  | *M* (*SD*) | 8.45 (4.71) |
|  | Minimal/ no anxiety (0-4) | 29 (26.13) |
|  | Mild (5-9) | 40 (36.04) |
|  | Moderate (10-14) | 30 (27.03) |
|  | Severe (≥ 15) | 12 (10.81) |
| PHQ-9 |  |  |
|  | *M* (*SD*) | 9.54 (5.90) |
|  | Minimal/ no depression (0-4) | 28 (25.23) |
|  | Mild (5-9) | 36 (32.43) |
|  | Moderate (10-14) | 20 (18.02) |
|  | Moderately severe (15-19) | 20 (18.02) |
|  | Severe (≥ 20) | 7 (6.31) |
| WCS |  |  |
|  | *M* (*SD*) | 33.53 (24.36) |
|  | High risk (≥ 57) | 22 (19.82) |
|  | Low risk (< 57) | 89 (80.18) |
| SITBI-G |  |  |
|  | Lifetime NSSI | 45 (40.54) |
|  | 12-month NSSI | 22 (19.82) |
|  |  |  |
|  | # of NSSI events (past 12 months) *M* (*SD*) (lifetime NSSI sample, *n* = 45) | 5.53 (16.06) |
|  | # of NSSI events (past 12 months) *M* (*SD*)  (12-month NSSI sample, *n* = 22) | 11.32 (21.72) |
|  | Age first NSSI (*n* = 45) | 14.13 (2.23) |
|  | Δ age last NSSI – age first NSSI (*n* = 45) | 3.07 (3.23) |
| AUDIT-C |  |  |
|  | *M* (*SD*) | 2.41 (1.72) |
|  | Abstinent (0) | 19 (17.12) |
|  | Moderate (1-3) | 63 (56.76) |
|  | Hazardous (≥ 4) | 29 (26.13) |
| Video acceptability |  |  |
|  | General likability | 3.90 (.71) |
|  | Comprehensibility | 4.87 (.33) |
|  | Interestingness | 3.97 (.80) |

## **Supplementary Table S2. Screening results and video acceptability of participants in the depression vignette group (*N* = 111)**

| Measure | Category | *M* (*SD*) or  *n* (%) |
| --- | --- | --- |
| GAD-7 |  |  |
|  | *M* (*SD*) | 9.10 (5.19) |
|  | Minimal/ no anxiety (0-4) | 23 (20.72) |
|  | Mild (5-9) | 41 (36.94) |
|  | Moderate (10-14) | 25 (22.52) |
|  | Severe (≥ 15) | 22 (19.82) |
| PHQ-9 |  |  |
|  | *M* (*SD*) | 10.15 (6.76) |
|  | Minimal/ no depression (0-4) | 30 (27.03) |
|  | Mild (5-9) | 28 (25.23) |
|  | Moderate (10-14) | 26 (23.42) |
|  | Moderately severe (15-19) | 13 (11.71) |
|  | Severe (≥ 20) | 14 (12.61) |
| WCS |  |  |
|  | *M* (*SD*) | 35.90 (25.20) |
|  | High risk (≥ 57) | 21 (18.92) |
|  | Low risk (< 57) | 90 (81.08) |
| SITBI-G |  |  |
|  | Lifetime NSSI | 52 (46.85) |
|  | 12-month NSSI | 30 (27.03) |
|  |  |  |
|  | # of NSSI events (past 12 months) *M* (*SD*) (lifetime NSSI sample, *n* = 52) | 27.69 (84.96) |
|  | # of NSSI events (past 12 months) *M* (*SD*)  (12-month NSSI sample, *n* = 30) | 48 (108.10) |
|  | Age first NSSI (*n* = 52) | 13.73 (2.31) |
|  | Δ age last NSSI – age first NSSI (*n* = 52) | 4.17 (3.31) |
| AUDIT-C |  |  |
|  | *M* (*SD*) | 2.86 (2.37) |
|  | Abstinent (0) | 26 (23.42) |
|  | Moderate (1-3) | 44 (39.64) |
|  | Hazardous (≥ 4) | 41 (36.94) |
| Video acceptability |  |  |
|  | General likability | 3.98 (.79) |
|  | Comprehensibility | 4.75 (.58) |
|  | Interestingness | 3.86 (.89) |

## **Supplementary Table S3. Screening results and video acceptability of participants in the Bulimia Nervosa vignette group (*N* = 108)**

| Measure | Category | *M* (*SD*) or  *n* (%) |
| --- | --- | --- |
| GAD-7 |  |  |
|  | *M* (*SD*) | 8.83 (5.09) |
|  | Minimal/ no anxiety (0-4) | 26 (24.07) |
|  | Mild (5-9) | 37 (34.26) |
|  | Moderate (10-14) | 29 (26.85) |
|  | Severe (≥ 15) | 16 (14.81) |
| PHQ-9 |  |  |
|  | *M* (*SD*) | 10.60 (6.38) |
|  | Minimal/ no depression (0-4) | 22 (20.37) |
|  | Mild (5-9) | 29 (26.85) |
|  | Moderate (10-14) | 25 (23.15) |
|  | Moderately severe (15-19) | 22 (20.37) |
|  | Severe (≥ 20) | 10 (9.26) |
| WCS |  |  |
|  | *M* (*SD*) | 37.19 (26.45) |
|  | High risk (≥ 57) | 23 (21.30) |
|  | Low risk (< 57) | 85 (78.70) |
| SITBI-G |  |  |
|  | Lifetime NSSI | 38 (35.19) |
|  | 12-month NSSI | 22 (20.37) |
|  |  |  |
|  | # of NSSI events (past 12 months) *M* (*SD*) (lifetime NSSI sample, *n* = 38) | 9.37 (21.13) |
|  | # of NSSI events (past 12 months) *M* (*SD*)  (12-month NSSI sample, *n* = 22) | 16.18 (25.90) |
|  | Age first NSSI (*n* = 38) | 13.79 (3.01) |
|  | Δ age last NSSI – age first NSSI (*n* = 38) | 3.92 (3.24) |
| AUDIT-C |  |  |
|  | *M* (*SD*) | 2.56 (2.11) |
|  | Abstinent (0) | 27 (25.00) |
|  | Moderate (1-3) | 44 (40.74) |
|  | Hazardous (≥ 4) | 37 (34.26) |
| Video acceptability |  |  |
|  | General likability | 3.79 (.77) |
|  | Comprehensibility | 4.87 (.34) |
|  | Interestingness | 3.89 (.96) |

## **Supplementary Table S4. Screening results and video acceptability of participants in the Non-Suicidal Self-Injury vignette group (*N* = 112)**

| Measure | Category | *M* (*SD*) or  *n* (%) |
| --- | --- | --- |
| GAD-7 |  |  |
|  | *M* (*SD*) | 8.35 (5.22) |
|  | Minimal/ no anxiety (0-4) | 33 (29.46) |
|  | Mild (5-9) | 41 (36.61) |
|  | Moderate (10-14) | 21 (18.75) |
|  | Severe (≥ 15) | 17 (15.18) |
| PHQ-9 |  |  |
|  | *M* (*SD*) | 9.61 (6.00) |
|  | Minimal/ no depression (0-4) | 22 (19.64) |
|  | Mild (5-9) | 39 (34.82) |
|  | Moderate (10-14) | 31 (27.68) |
|  | Moderately severe (15-19) | 11 (9.82) |
|  | Severe (≥ 20) | 9 (8.04) |
| WCS |  |  |
|  | *M* (*SD*) | 33.75 (23.13) |
|  | High risk (≥ 57) | 23 (20.54) |
|  | Low risk (< 57) | 89 (79.46) |
| SITBI-G |  |  |
|  | Lifetime NSSI | 30 (26.79) |
|  | 12-month NSSI | 14 (12.50) |
|  |  |  |
|  | # of NSSI events (past 12 months) *M* (*SD*) (lifetime NSSI sample, *n* = 30) | 5.10 (10.30) |
|  | # of NSSI events (past 12 months) *M* (*SD*)  (12-month NSSI sample, *n* = 14) | 10.93 (12.96) |
|  | Age first NSSI (*n* = 30) | 13.60 (3.59) |
|  | Δ age last NSSI – age first NSSI (*n* = 30) | 4.63 (3.91) |
| AUDIT-C |  |  |
|  | *M* (*SD*) | 2.55 (2.30) |
|  | Abstinent (0) | 27 (24.11) |
|  | Moderate (1-3) | 52 (46.43) |
|  | Hazardous (≥ 4) | 33 (29.46) |
| Video acceptability |  |  |
|  | General likability | 3.79 (.83) |
|  | Comprehensibility | 4.77 (.50) |
|  | Interestingness | 3.87 (.95) |

## **Supplementary Table S5. Screening results and video acceptability of participants in the problematic alcohol use vignette group (*N* = 112)**

| Measure | Category | *M* (*SD*) or  *n* (%) |
| --- | --- | --- |
| GAD-7 |  |  |
|  | *M* (*SD*) | 8.30 (5.12) |
|  | Minimal/ no anxiety (0-4) | 36 (32.14) |
|  | Mild (5-9) | 32 (28.57) |
|  | Moderate (10-14) | 28 (25.00) |
|  | Severe (≥ 15) | 16 (14.29) |
| PHQ-9 |  |  |
|  | *M* (*SD*) | 8.95 (5.67) |
|  | Minimal/ no depression (0-4) | 33 (29.46) |
|  | Mild (5-9) | 31 (27.68) |
|  | Moderate (10-14) | 28 (25.00) |
|  | Moderately severe (15-19) | 15 (13.39) |
|  | Severe (≥ 20) | 5 (4.46) |
| WCS |  |  |
|  | *M* (*SD*) | 31.96 (22.01) |
|  | High risk (≥ 57) | 17 (15.18) |
|  | Low risk (< 57) | 95 (84.82) |
| SITBI-G |  |  |
|  | Lifetime NSSI | 38 (33.93) |
|  | 12-month NSSI | 25 (22.32) |
|  |  |  |
|  | # of NSSI events (past 12 months) *M* (*SD*) (lifetime NSSI sample, *n* = 38) | 19.42 (55.21) |
|  | # of NSSI events (past 12 months) *M* (*SD*)  (12-month NSSI sample, *n* = 25) | 29.52 (66.25) |
|  | Age first NSSI (*n* = 38) | 13.84 (2.65) |
|  | Δ age last NSSI – age first NSSI (*n* = 37)* | 4.16 (3.35) |
| AUDIT-C |  |  |
|  | *M* (*SD*) | 2.54 (1.89) |
|  | Abstinent (0) | 20 (17.86) |
|  | Moderate (1-3) | 58 (51.79) |
|  | Hazardous (≥ 4) | 34 (30.36) |
| Video acceptability |  |  |
|  | General likability | 3.79 (.79) |
|  | Comprehensibility | 4.69 (.63) |
|  | Interestingness | 3.63 (1.02) |

**n* = 1 missing due to invalid values (age of first NSSI > age of last NSSI)
